# TAZ/NRF2 positive feedback loop contributes to proliferation in bladder cancer through antagonistic ferroptosis

**DOI:** 10.1038/s41420-025-02506-9

**Published:** 2025-04-29

**Authors:** Zhen Song, Shikai Gui, Xuepeng Rao, Gan Zhang, Yu Cheng, Tao Zeng

**Affiliations:** 1 Department of Urology, The Second Affiliated Hospital, Jiangxi Medical College, Nanchang University, Nanchang, Jiangxi 330006 China; 2 Department of Neurosurgery, The Second Affiliated Hospital, Jiangxi Medical College, Nanchang University, Nanchang, Jiangxi 330006 China

**Keywords:** Bladder cancer, Cell death

## Abstract

Bladder cancer (BLCA) is a prevalent malignancy characterized by high recurrence and metastasis rates. Emerging evidence suggests that the NRF2-GPX4 axis is closely associated with ferroptosis. The transcriptional coactivator with PDZ-binding motif (TAZ) plays a crucial role in regulating ferroptosis; however, its role in BLCA remains unclear. In our study, we found that TAZ was markedly upregulated in BLCA tissues and BLCA cell lines. Gene set enrichment analysis indicated that TAZ depletion was related to ferroptosis and glutathione metabolism. Our results demonstrated that TAZ promotes the malignant progression of BLCA cells both in vitro and in vivo. Moreover, TAZ enhances NRF2 transcriptional activity through interaction with NRF2. We further revealed that TAZ-TEAD4 regulates NRF2 expression at the transcriptional level. Additionally, NRF2 regulates TAZ transcription by binding to its promoter region, establishing a positive feedback loop between TAZ and NRF2 that sustains GPX4 activation and inhibits ferroptosis in BLCA. These insights provide novel molecular targets for therapeutic treatment in BLCA.

## Introduction

Bladder cancer (BLCA) ranks as the fourth most common cancer globally and is influenced by various genetic and environmental risk factors that drive its development and progression [1, 2]. BLCA can be classified into non-muscle-invasive bladder cancer (NMIBC), which accounts for 75% of cases, and muscle-invasive bladder cancer (MIBC) or metastatic disease, accounting for the remaining 25% [3]. Notably, up to 45% of NMIBC patients may experience progression to MIBC, which is associated with a poor prognosis [4]. Therefore, investigation of the biological mechanisms underlying BLCA progression is crucial for developing effective therapeutic strategies for BLCA patients.

Ferroptosis is regulated by various cellular metabolic pathways, including iron homeostasis, redox balance, mitochondrial activity, and the metabolism of amino acids, carbohydrates, and lipids [5, 6]. This process is implicated in multiple physiological conditions and various diseases, particularly cancers [7, 8]. The mechanisms that govern ferroptosis are complex and involve several transcription factors, such as ATF4, p53, NUPR1, and nuclear factor erythroid 2-related factor 2 (NRF2). As a key transcriptional regulator of cellular redox homeostasis, NRF2 protects cells from ferroptotic death [9, 10]. It reduces oxidative stress and lipid peroxidation by inducing antioxidant response element (ARE)-containing genes, including glutathione peroxidase 4 (GPX4) [11]. For example, the activation of the NRF2 pathway enhances GPX4 levels, thereby inhibiting ferroptosis induced by sorafenib in hepatocellular carcinoma [12]. Furthermore, NRF2 confers resistance to EGFR tyrosine kinase inhibitors by upregulating GPX4 in lung cancer [13]. Therefore, targeting ferroptosis represents a promising therapeutic approach in bladder cancer.

The Hippo pathway is a highly conserved regulatory system involved in controlling organ size, regeneration, and tissue homeostasis [14, 15]. The transcriptional coactivators YAP1 and TAZ serve as pivotal effectors in this pathway, interacting with TEAD1–4 to regulate genes involved in stem cell self-renewal, apoptosis, and cell proliferation [16, 17]. Emerging evidence indicates that dysregulation of the Hippo pathway in human cancers is linked to ferroptosis and may promote uncontrolled cell growth and malignant transformation [18, 19]. For example, Gao et al. emphasized the critical role of YAP/TAZ in repressing ferroptosis in hepatocellular carcinoma [20]. In breast cancer, the YAP/TAZ-mediated regulation of LAMC2 confers resistance to ferroptosis in conjunction with β4 integrin [21]. However, the role of TAZ in the regulation of ferroptosis remains unclear in bladder cancer.

In this study, we demonstrated that TAZ was overexpressed in BLCA tissues and cell lines. GSEA suggested that TAZ depletion was associated with ferroptosis and glutathione metabolism. Overexpression of TAZ promotes cell proliferation and inhibits ferroptosis by activating the NRF2/GPX4 pathway in BLCA. Moreover, we discovered that NRF2 maintains GPX4 upregulation by establishing a positive feedback loop with TAZ. Our results revealed that the TAZ/NRF2 positive feedback loop inhibited ferroptosis in bladder cancers by regulating GPX4, offering a novel therapeutic potential for patients with BLCA.

## Results

### TAZ is highly expressed in BLCA

**Fig. 1:**
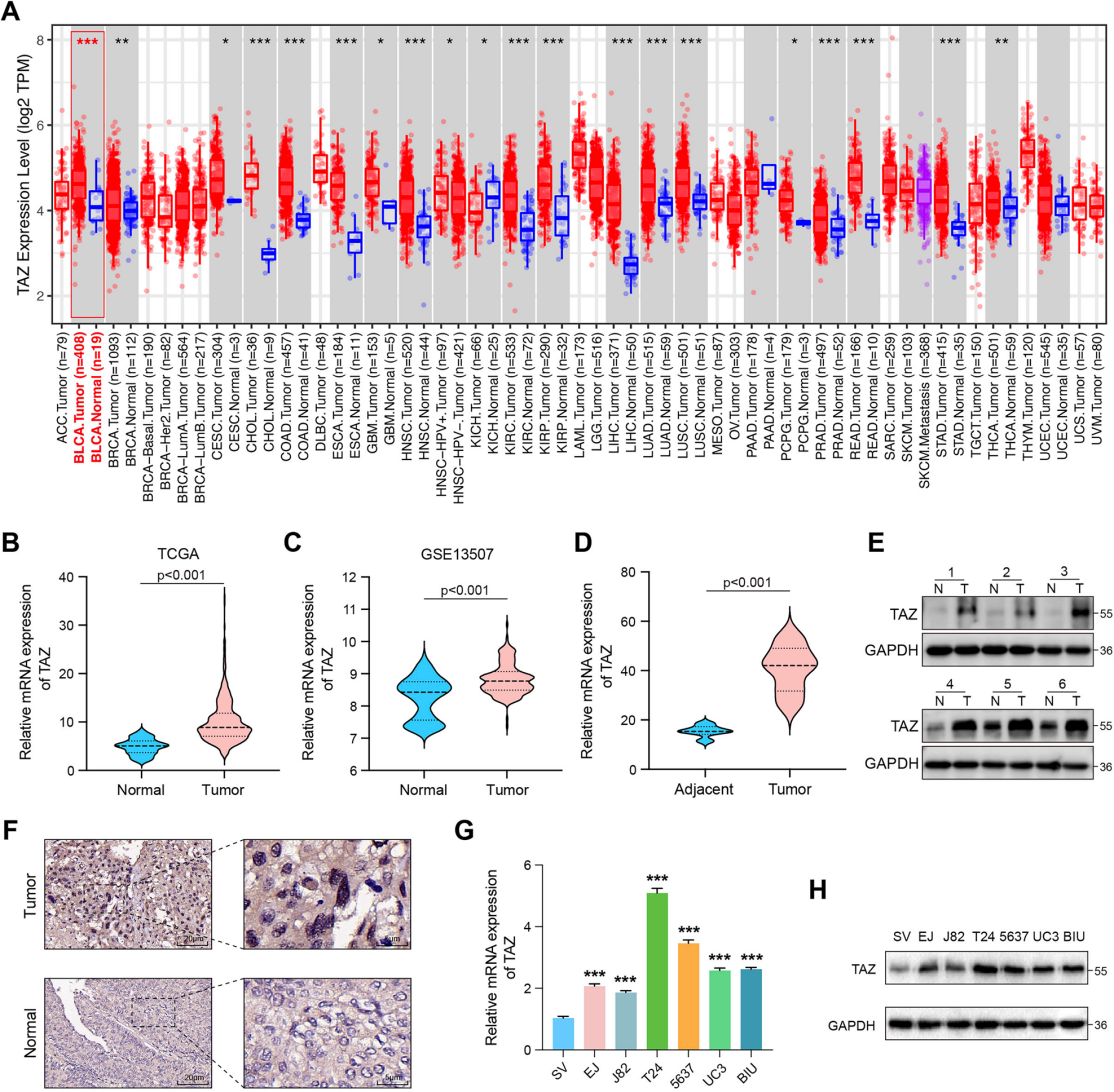
TAZ is high expression in BLCA. **A** The differential expression of TAZ in diverse tumor tissues and their respective normal tissues. **B** TAZ expression levels in bladder cancer tissue and normal tissue in the TCGA database. **C** The differential expression of TAZ in the GSE13507 database. **D**, **E** mRNA and protein expression levels of TAZ in bladder cancer tissue and adjacent normal tissue. **F** The expression of TAZ was determined in BLCA specimens and normal samples using immunohistochemistry. **G**, **H** qRT-PCR and western blot analysis of TAZ expression in BLCA cell lines. *p < 0.05, **p < 0.01, ***p < 0.001.

We first examined TAZ expression across various cancers using the TIMER2 database, which revealed elevated TAZ levels in several malignancies, including BLCA, esophageal cancer (ESCA), breast cancer (BRCA), and glioblastoma (GBM) (Fig. 1A). A combined analysis of the TCGA dataset and the GSE13507 dataset showed that TAZ was upregulated in BLCA (Fig. 1B, C). We then validated both mRNA and protein levels in BLCA samples and found that TAZ was significantly overexpressed in BLCA tissues compared to adjacent normal tissues (Fig. 1D, E). Immunohistochemical staining further demonstrated higher TAZ expression in BLCA tissues (Fig. 1F). Additionally, TAZ expression was elevated in BLCA cell lines compared to normal human uroepithelial cells SV-HUC-1 (Fig. 1G, H). Overall, these findings indicated that TAZ was upregulated in BLCA.

### TAZ promotes malignant phenotypes of BLCA cells both in vitro and in vivo

**Fig. 2:**
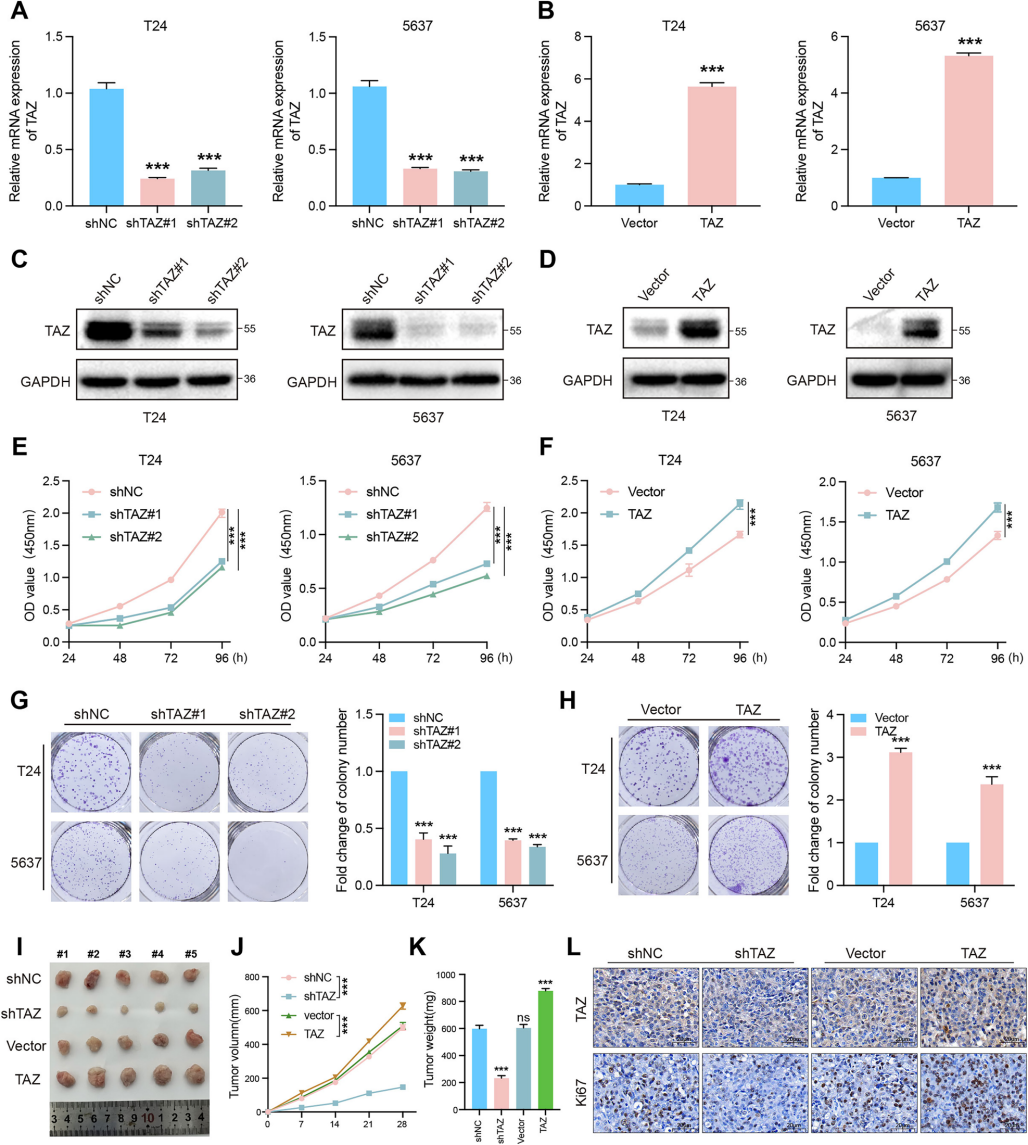
TAZ promotes malignant phenotypes of BLCA cells both in vitro and in vivo. **A**–**D** qRT-PCR and western blot analyses were performed to confirm the efficiency of TAZ knockdown and overexpression in T24 and 5637 cells. **E** CCK-8 assay was performed to assess cell viability in control and TAZ knockdown cells. **F** CCK-8 assay was performed to assess cell viability in control and TAZ-overexpressing cells. **G**, **H** The effect of TAZ expression levels on colony formation in T24 and 5637 cells was assessed using colony formation assays. **I** The subcutaneous xenograft mouse model showed that both knockdown and overexpression of TAZ affect the growth of BLCA cells in vivo (*n* = 5, *P*-value < 0.05). **J** Tumor volume and **K** tumor weight were determined. **L** Immunofluorescence was implemented to detect the expression levels of Ki67 using tumor tissues harvested from xenograft model mice. *p < 0.05, **p < 0.01, ***p < 0.001.

To explore the biological function of TAZ in BLCA, we established TAZ-silenced and TAZ-overexpressing T24 and 5637 BLCA cell lines. The efficiency of knockdown and overexpression was validated by qRT-PCR and western blot (Fig. 2A–D). CCK-8 assays revealed that TAZ knockdown suppressed cell proliferation, while TAZ overexpression significantly enhanced this ability (Fig. 2E, F). Colony formation assays further corroborated that TAZ depletion led to a reduction in colony formation, whereas TAZ overexpression had a reversed effect (Fig. 2G, H). Additionally, we selected the J82 cell line, which exhibits low TAZ expression, for further analysis. CCK-8 and colony formation assays further indicated that TAZ knockdown significantly inhibited cell proliferation, whereas TAZ overexpression enhanced the proliferative capacity of J82 cells (Fig. S1A–D). To investigate whether TAZ facilitates the proliferation of BLCA cells in vivo, T24 cells transfected with their corresponding lentiviral vectors were subsequently injected into nude mice. The results indicated that TAZ knockdown resulted in smaller tumors and lower tumor weights, whereas TAZ overexpression promoted tumor growth (Fig. 2I–K). Immunohistochemical staining confirmed that TAZ overexpression enhanced cell proliferation and increased Ki67 levels (Fig. 2L). Collectively, these findings highlight the critical role of TAZ in promoting BLCA cell growth both in vitro and in vivo.

### TAZ facilitates the proliferation of BLCA cells by inhibiting ferroptosis

**Fig. 3:**
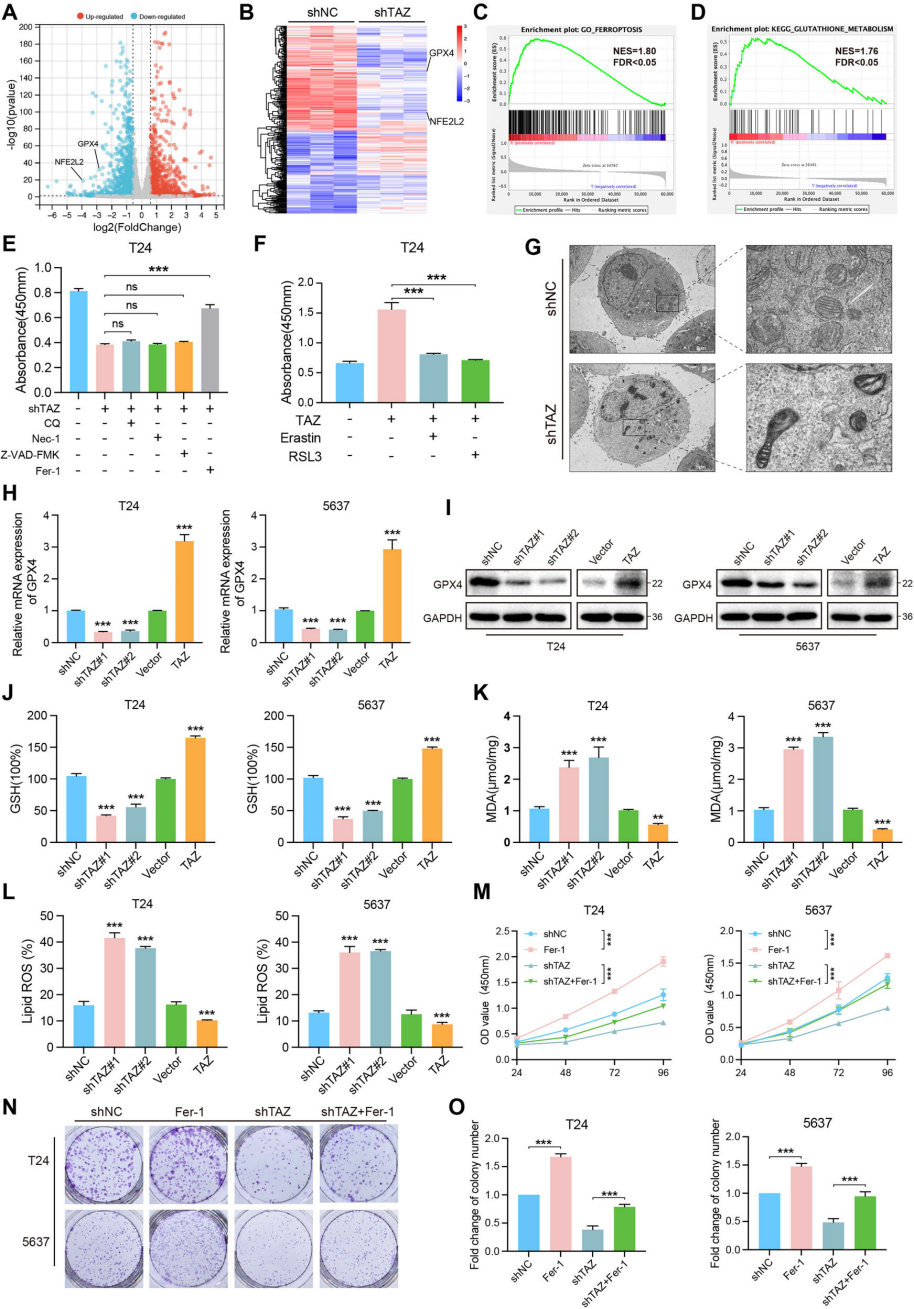
TAZ facilitates the proliferation of BLCA cells by inhibiting ferroptosis. **A**, **B** Volcano plots and heatmaps of RNA-sequencing. **C**, **D** GSEA results indicated that TAZ knockdown was closely associated with ferroptosis and glutathione metabolism. **E** The effect on cell viability was assessed using Nec-1, CQ, Z-VAD-FMK, and Fer-1 in the TAZ knockdown T24 cell line. **F** Erastin and RSL3 were used to assess the effect on cell viability in the T24 cell line overexpressing TAZ. **G** Mitochondrial changes in different groups. **H**, **I** qPCR and Western blotting assays were conducted to verify GPX4 expression following TAZ knockdown and overexpression. **J**, **K** MDA and GSH levels were detected in the different groups. **L** Flow cytometry analysis of intracellular ROS levels in T24 and 5637 cells in the different groups. **M** CCK-8 assay was conducted at 24, 48, 72, and 96 h using T24 and 5637 cells from different groups (Vector, Fer-1, shTAZ, and shTAZ+ Fer-1). **N**, **O** Colony formation assays were performed to assess the effects of Fer-1 treatment on colony formation in TAZ-knockdown T24 and 5637 cells. *p < 0.05, **p < 0.01, ***p < 0.001.

To explore the molecular mechanisms by which TAZ influences the progression of BLCA, RNA-seq analysis was performed on T24-shNC and T24-shTAZ cells. The differentially expressed genes, both downregulated and upregulated, were shown in the Volcano plot and heatmap (Fig. 3A, B), including NFE2L2 and GPX4. Then, GSEA results indicated that TAZ depletion was associated with ferroptosis and glutathione metabolism (Fig. 3C, D). The glutathione metabolic process is closely associated with ferroptosis [22]. To identify the specific type of cell death affected by TAZ knockdown, we employed inhibitors targeting necroptosis (Nec-1), autophagy (CQ), apoptosis (Z-VAD-FMK), and ferroptosis (Fer-1). Notably, only Fer-1 reversed the inhibitory effect of TAZ knockdown on cell growth (Fig. 3E), while ferroptosis-inducing agents Erastin and RSL3 inhibited the growth of TAZ-overexpressing cells (Fig. 3F). Transmission electron microscopy (TEM) revealed more pronounced mitochondrial damage in TAZ-depleted T24 cells, characterized by shrunken mitochondria and ruptured cristae (Fig. 3G). These results suggested that TAZ inhibits ferroptosis in BLCA cells.

Emerging evidence suggests that GPX4 plays a crucial role in the glutathione metabolic process [23]. Consequently, we hypothesized that TAZ may be involved in the modulation of ferroptosis by influencing GPX4 expression. However, western blotting experiments revealed that the protein levels of SLC7A11, TRFC, and FLT did not change significantly following TAZ expression reduction. In contrast, GPX4 protein levels were significantly downregulated (Fig. S2A). Subsequently, we assessed both mRNA and protein levels of GPX4, finding that TAZ depletion resulted in decreased GPX4 expression, whereas TAZ overexpression markedly increased GPX4 levels (Fig. 3H, I). Knockdown of TAZ resulted in decreased levels of GSH, while TAZ overexpression led to increased levels of GSH (Fig. 3J). Conversely, TAZ knockdown was associated with elevated MDA levels, whereas overexpression of TAZ induced the reverse effect (Fig. 3K). We further measured reactive oxygen species (ROS) levels in BLCA cells, and our results demonstrated that TAZ inhibition elevated ROS levels, while TAZ overexpression significantly reduced ROS levels in BLCA cells (Fig. 3L). Furthermore, the CCK8 and colony formation assays indicated that Fer-1 could reverse the inhibitory effects of TAZ depletion on BLCA cell proliferation (Fig. 3M–O). Additionally, treatment with RSL3 reduced cell proliferation induced by TAZ overexpression (Fig. S2B, C). Collectively, these findings confirmed that TAZ regulates BLCA cell proliferation by inhibiting ferroptosis.

### TAZ interacts with NRF2

**Fig. 4:**
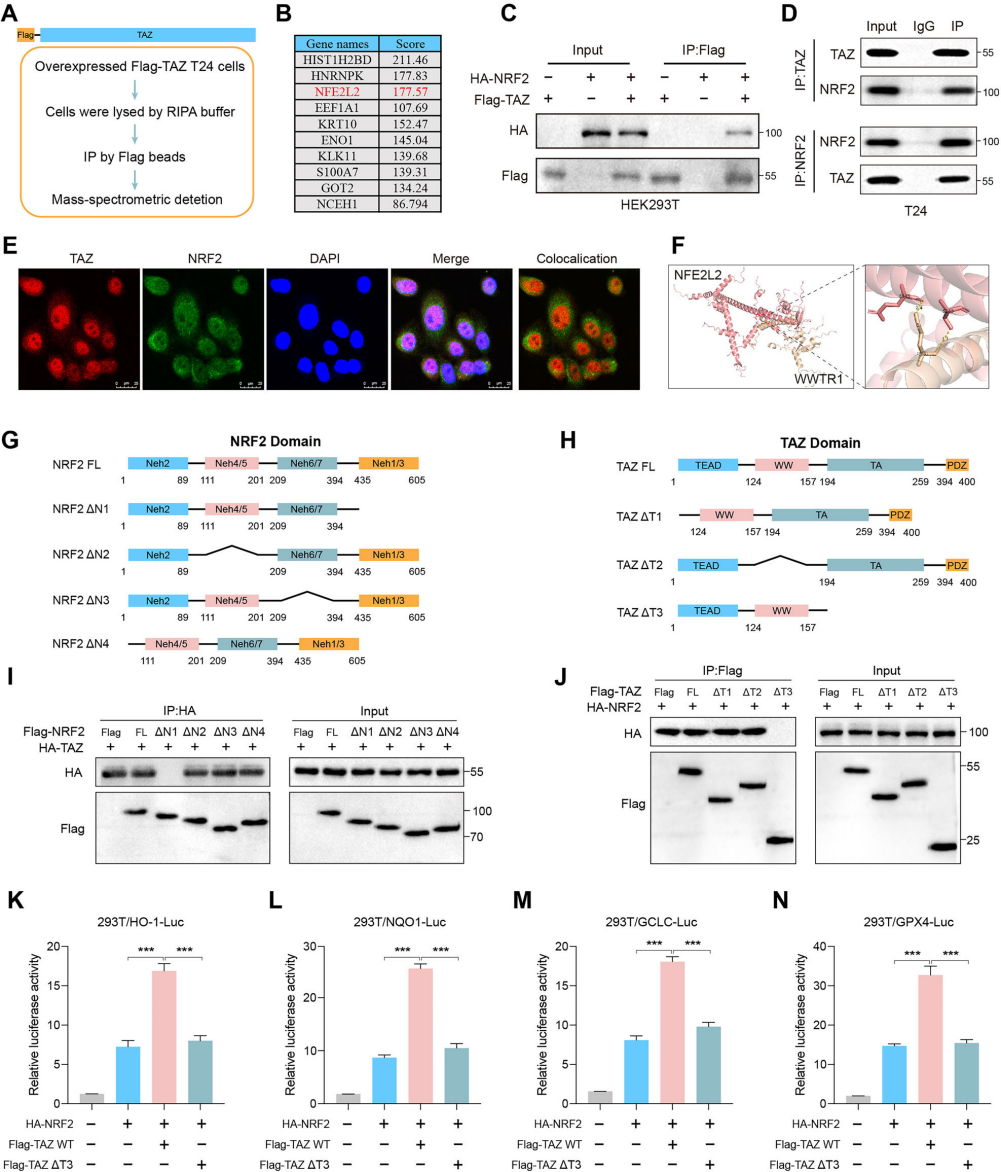
TAZ interacts with NRF2. **A** IP assays were conducted in T24 cells, followed by mass spectrometry for analysis and identification. **B** List of TAZ-associated proteins identified by mass spectrometric analysis. **C** Western blot of interaction between overexpressed HA-TAZ with Flag-NRF2 in HEK293T cells. **D** Western blot analysis was conducted to assess the interaction between endogenous TAZ and NRF2 in T24 cells. **E** Immunofluorescent staining of TAZ (green) and NRF2 (red) in T24 cells. Nuclear 4′, 6-diamidino-2-phenylindole (DAPI; blue). **F** The binding interface between TAZ and NRF2 was based on the molecular docking model. **G** Schematic structure of NRF2 truncations. **H** Schematic structure of TAZ truncations. **I** Western blot analysis of interaction between TAZ and NRF2 domain deleted mutants in HEK293T cells. **J** Western blot analysis of interaction between NRF2 and TAZ domain deleted mutants in HEK293T cells. **K**–**N** The TAZ Δ3 deletion mutants exhibited no significant effect on the transcriptional activity of NRF2 target genes (HO-1, NQO1, GCLC, GPX4) as measured by the Luc reporter assay. *p < 0.05, **p < 0.01, ***p < 0.001.

To elucidate the mechanisms underlying how TAZ promotes malignant progression of BLCA, we conducted immunoprecipitation followed by mass spectrometry analysis to identify TAZ-interacting proteins (Fig. 4A). Our results indicated that TAZ may interact with NRF2 protein in BLCA cells (Fig. 4B). To confirm this, NRF2 or TAZ-expressing plasmids were transfected separately or co-transfected with HEK293T cells, and NRF2 was detected in the immunoprecipitated complexes of TAZ (Fig. 4C). Additionally, the interaction between endogenous NRF2 and TAZ was confirmed by co-immunoprecipitation (Co-IP) (Fig. 4D). These findings showed that TAZ can bind to NRF2 under both endogenous and exogenous conditions, indicating a direct interaction between TAZ and NRF2. Immunofluorescence (IF) staining further illustrated that endogenous TAZ colocalized with NRF2 (Fig. 4E). Molecular docking model predicted that the TAD domain of TAZ interacts with the Neh1/3 domain of NRF2 (Fig. 4F–H). Moreover, Co-IP experiments confirmed that the Neh1/3 domain of NRF2 interacts with TAZ, while the TAD domain in TAZ was responsible for this interaction (Fig. 4I, J). NRF2 contains seven domains known as Neh1–Neh7, while the Neh3 domain was important for the transactivation activity of NRF2 [24]. To investigate whether the interaction between TAZ and NRF2 affects the transcriptional activity of NRF2, we introduced reporter genes containing the antioxidant response element (ARE) promoter, including HO-1-luc, NQO1-luc, GCLC-luc, and GPX4-luc. The results revealed that ectopic expression of TAZ significantly enhanced ARE promoter activity, whereas the deletion of the TAD domain of TAZ mitigated this effect (Fig. 4K–N). Our findings indicated that TAZ interacts with NRF2 and enhances its transcriptional activity.

### TAZ regulates NRF2 expression at the transcriptional level

**Fig. 5:**
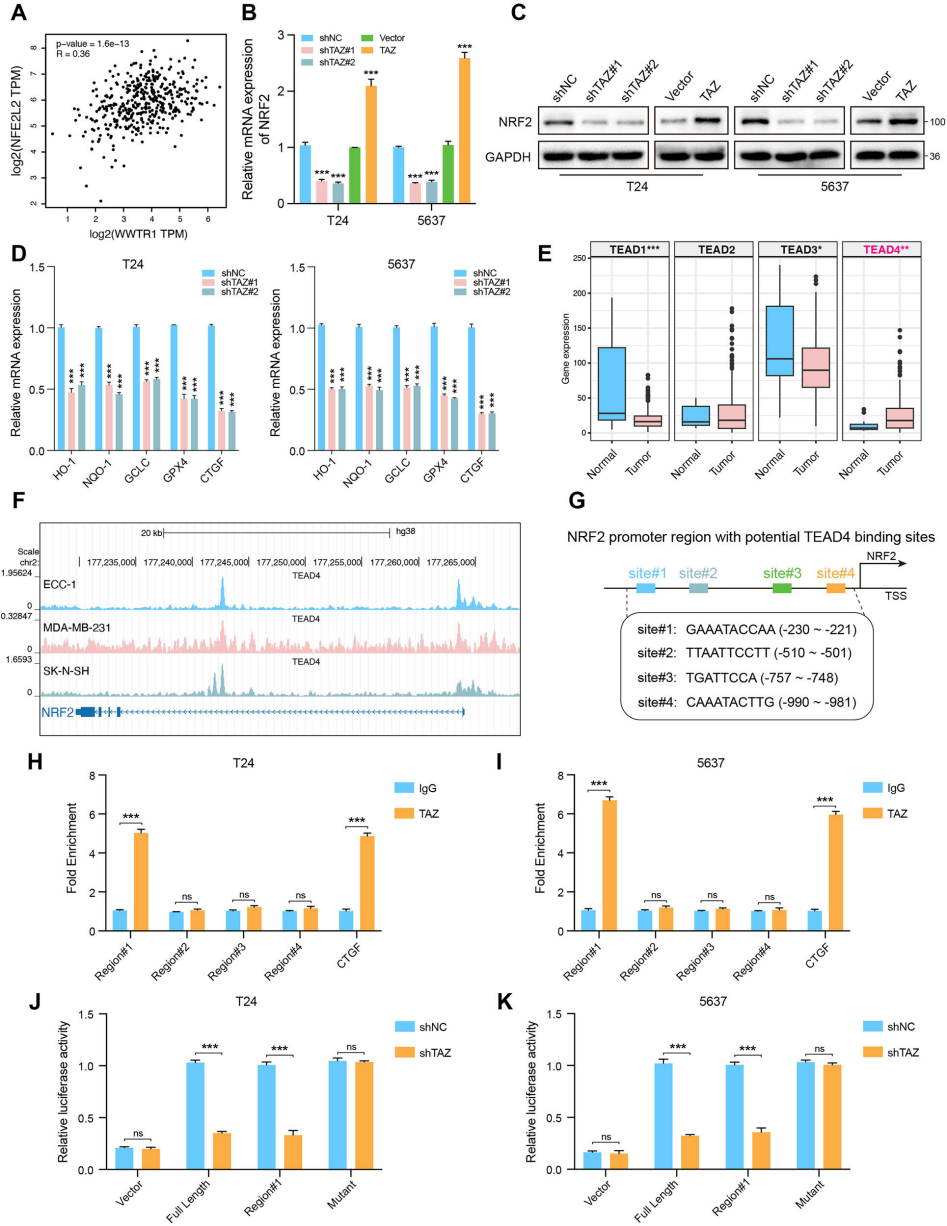
TAZ regulates NRF2 expression at the transcriptional level. **A** Correlation between TAZ and NRF2 in the TCGA BLCA database. **B**, **C** qRT-PCR and western blot analyses were used to detect GPX4 expression in T24 and 5637 cells with TAZ overexpression and TAZ knockdown. **D** Expression level of downstream target genes of NRF2. **E** TEAD1-4 expression levels in bladder cancer tissue and normal tissue in the TCGA database. **F** Schematic diagram of the binding motif of the transcription factor TEAD4. **G** Schematic diagram of potential TEAD4 binding sites in the promoter region of NRF2. **H**, **I** ChIP-qPCR analysis was performed in T24 and 5637 cells to evaluate the enrichment of TAZ on the NRF2 promoter, and IgG was used as a negative control. **J**, **K** The luciferase activity of wild-type and mutant NRF2 promoters was measured in T24 and 5637 cells. *p < 0.05, **p < 0.01, ***p < 0.001.

Spearman correlation analysis revealed a positive association between TAZ and NRF2 expression (Fig. 5A). Then, we examined whether TAZ regulates NRF2 at the transcriptional level. The results indicated that both mRNA and protein levels of NRF2 decreased significantly following TAZ knockdown, whereas they increased after TAZ overexpression (Fig. 5B, C). To assess the impact of TAZ on NRF2 transcriptional activity, we measured the mRNA levels of downstream target genes of NRF2 following TAZ knockdown. Our results demonstrated that silencing TAZ led to a reduction in the expression levels of NRF2 target genes, including GPX4 (Fig. 5D), suggesting that TAZ can transcriptionally regulate NRF2 expression. Due to the absence of DNA-binding domains, TAZ typically functions as a transcriptional coactivator and has been shown to interact with various transcription factors, particularly TEAD transcription factors [25]. By analyzing all TEAD family members in TCGA BLCA dataset, we found that TEAD4 was the most highly expressed in BLCA (Fig. 5E). Furthermore, we analyzed publicly available ChIP-seq data from the Cistrome database (http://cistrome.org/db/#/), which revealed that TEAD4 binds to the promoter regions of NRF2 (Fig. 5F). Thus, we examined potential TEAD4 binding sites in the NRF2 promoter using the JASPAR (https://jaspar.elixir.no/) database and identified four conserved sites (Fig. 5G). The ChIP-qPCR results showed that TAZ binds to the NRF2 promoter at site 1 (Fig. 5H, I). Additionally, dual-luciferase reporter assays indicated that overexpression of TAZ inhibited the transcriptional activity of the NRF2 promoter. Notably, mutations in NRF2 (site1) almost completely abolished this effect (Fig. 5J, K). Taken together, these findings confirmed that NRF2 was regulated by TAZ at the transcriptional level.

### TAZ inhibits ferroptosis and promotes BLCA proliferation by activating NRF2

**Fig. 6:**
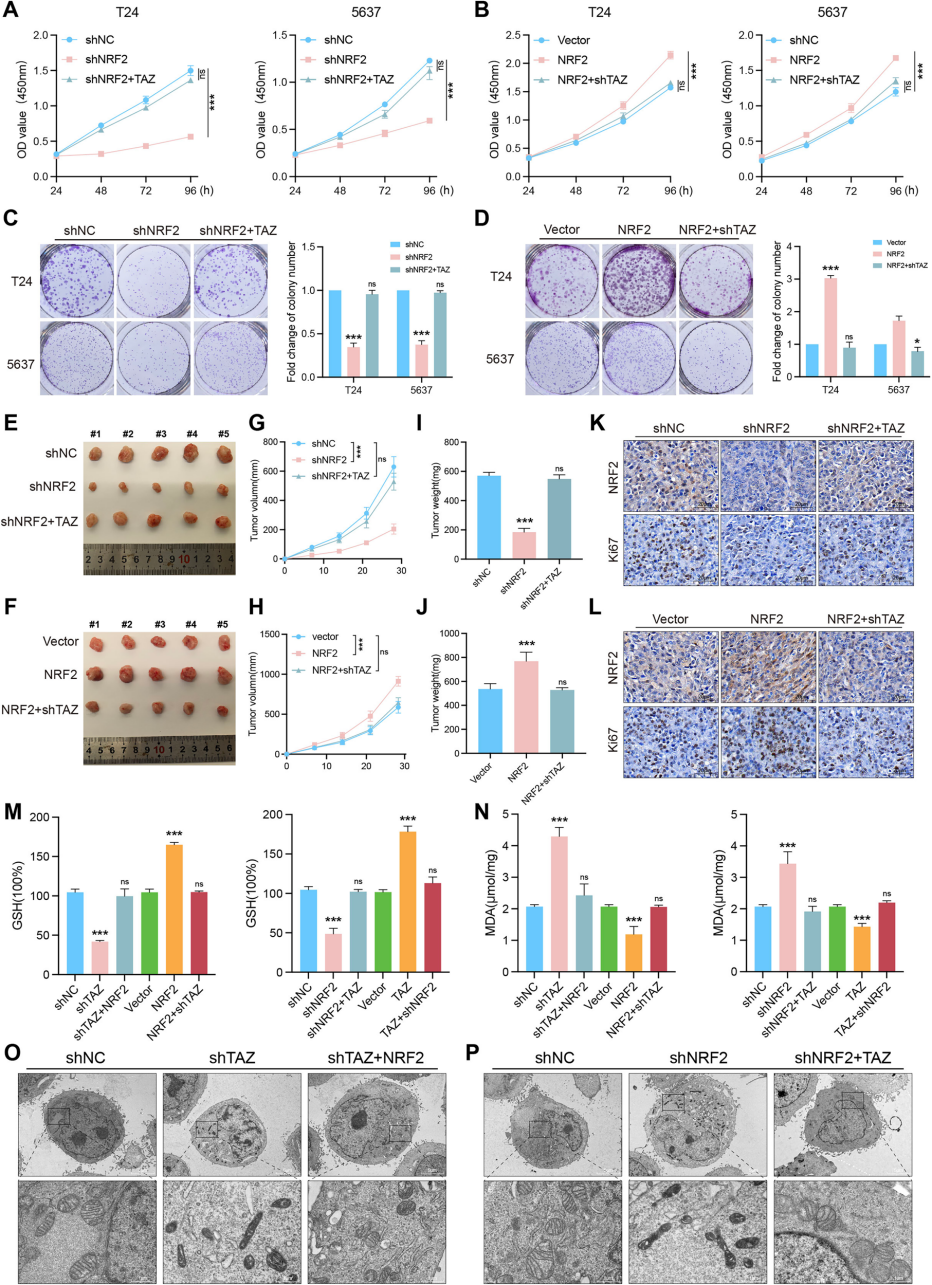
TAZ inhibits ferroptosis and promotes BLCA proliferation by activating NRF2. NRF2 knockdown inhibited BLCA cells’ proliferation, which was rescued by TAZ overexpression as indicated by CCK-8 (**A**, **B**), colony formation (**C**, **D**). Equal numbers of different T24 cell populations (T24-shNC, T24-shNRF2, T24-shNRF2+TAZ, T24-Vector, T24-NRF2, T24-NRF2+shTAZ) were used to establish subcutaneous xenograft tumors. The tumors were harvested and photographed (*n* = 5 each group). **E**–**L** The subcutaneous xenograft mouse model showed that both knockdown and overexpression of NRF2 affect the growth of BLCA cells in vivo, while knockdown and overexpression of TAZ reversed these effects (*n* = 5, *P*-value < 0.05). **G**, **H** Tumor volume and **I**, **J** tumor weight were determined. **K**, **L** Immunofluorescence was implemented to detect the expression levels of Ki67 using tumor tissues harvested from xenograft model mice. **M**, **N** MDA and GSH levels were detected in the different groups of treated T24 and 5637 cells. **O**, **P** Mitochondrial changes in different groups. *p < 0.05, **p < 0.01, ***p < 0.001.

To explore the role of NRF2 in BLCA, we transfected knockdown or overexpression lentivirus vectors of NRF2 into T24 and 5637 cells (Fig. S3A–D). Colony formation and CCK8 assays suggested that NRF2 knockdown suppressed the proliferation of BLCA cells, which was rescued by TAZ overexpression (Fig. 6A–D). Then, we established a xenograft mouse model to examine whether the correlation between NRF2 and TAZ contributed to BLCA progression in vivo. The mice were randomly divided into six groups and injected with T24-shNC, T24-shNRF2, T24-shNRF2+TAZ, T24-Vector, T24-NRF2, and T24-NRF2+shTAZ cells. Throughout the feeding period, tumor volume and weight were regularly monitored. Compared to the shNC group, mice in the shNRF2 group exhibited reduced tumor volume and weight, while overexpression of TAZ partially mitigated these effects (Fig. 6E–J). Ki67 immunostaining further confirmed that NRF2 knockdown reduced tumor proliferation, and this effect was rescued by TAZ overexpression (Fig. 6K). Notably, overexpression of NRF2 promoted tumor growth and increased tumor volume, tumor weight, and Ki-67 expression levels, while depletion of TAZ reversed these effects (Fig. 6L). Additionally, our findings showed that NRF2 overexpression promoted tumor growth and increased tumor volume, tumor weight, and Ki-67 expression levels, while treatment with RSL3 reversed these effects (Fig. S3E–H). Furthermore, GSH levels were decreased in the shNRF2 group and increased in the NRF2 overexpression group, with TAZ depletion reversing this trend (Fig. 6M). NRF2 reduced the levels of MDA, which could be rescued by depletion of TAZ (Fig. 6N). As expected, TEM analysis revealed shrunken mitochondria with ruptured cristae and increased membrane density in NRF2-depleted cells, which was reversed upon the ectopic expression of TAZ (Fig. 6O, P). Taken together, these results demonstrated that TAZ promotes BLCA cell proliferation and inhibits ferroptosis through transcriptionally activating NRF2.

### TAZ sustains the activation of GPX4-mediated ferroptosis resistance by establishing a positive feedback loop with NRF2

**Fig. 7:**
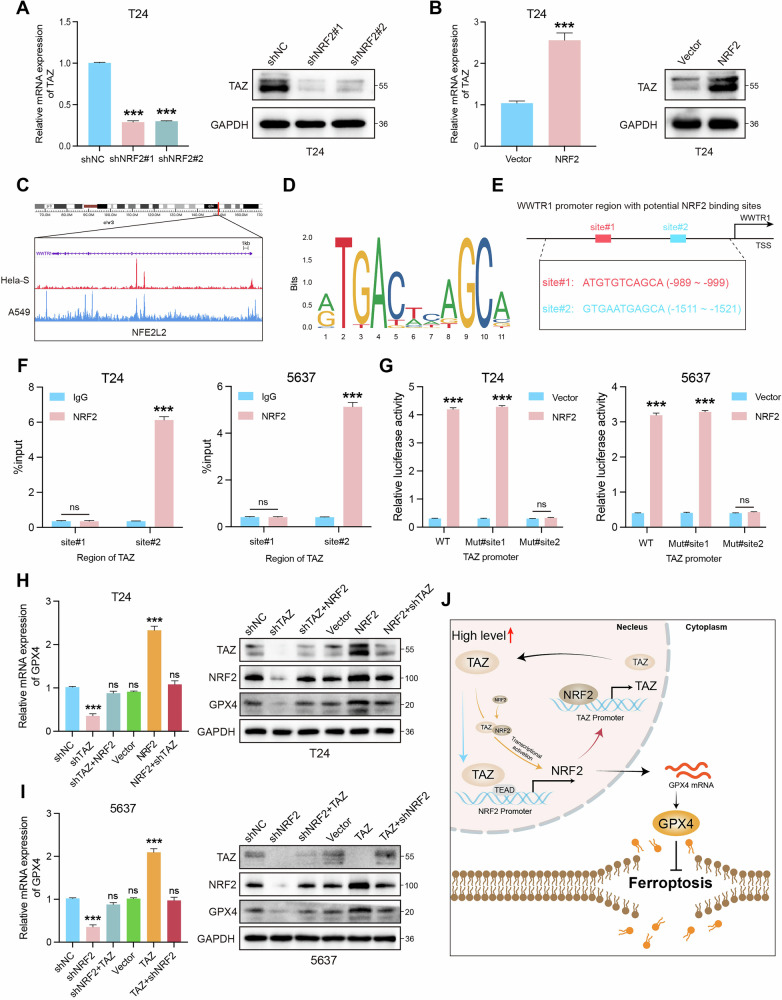
TAZ sustains the activation of GPX4-mediated ferroptosis resistance by establishing a positive feedback loop with NRF2. **A**, **B** The mRNA and protein expression levels of TAZ. **C** The publicly available ChIP-seq data showed that NRF2 binds to the promoter regions of TAZ. **D** Schematic diagram of the binding motif of the transcription factor NRF2. **E** Schematic diagram of potential NRF2 binding sites in the promoter region of TAZ. **F** ChIP-qPCR analysis was performed in T24 and 5637 cells to evaluate the enrichment of NRF2 on the TAZ promoter, and IgG was used as a negative control. **G** The luciferase activity of wild-type and mutant NRF2 promoters was measured in T24 and 5637 cells. **H**, **I** qPCR and Western blotting assays were conducted to verify GPX4 expression in each experimental group. **J** Schematic diagram of the molecular mechanism of this study. *p < 0.05, **p < 0.01, ***p < 0.001.

Interestingly, we unexpectedly discovered that NRF2 depletion reduced the protein and mRNA expression levels of TAZ (Fig. 7A, B). Subsequently, we introduced Verteporfin, a TAZ inhibitor, to evaluate TAZ expression in NRF2-overexpressing BLCA cells compared to control vector cells. The results indicated that TAZ expression levels were lower in the Verteporfin-treated group than in the NRF2 overexpressing group (Fig. S4A). CCK8 and colony formation assays indicated that treatment with Verteporfin partially abolished the proliferation ability induced by NRF2 overexpression (Fig. S4B, C). Moreover, the application of ML385, an NRF2 inhibitor, resulted in decreased NRF2 expression levels compared to the TAZ overexpression group (Fig. S4D). Notably, inhibiting NRF2 with ML385 reversed the malignant progression of BLCA cells induced by TAZ overexpression (Fig. S4E, F). A recent study indicates that NRF2 regulates TAZ through transcriptional activation [26]. Subsequently, we analyzed publicly available ChIP-seq data from the Cistrome database (http://cistrome.org/db/#/), and results found that NRF2 binds to the promoter regions of TAZ (Fig. 7C, D). Thus, we inferred that NRF2 might regulate TAZ at the transcriptional level. To identify potential NRF2 binding sites, we screened the TAZ promoter region using the JASPAR database, and two conserved binding sites were identified (Fig. 7E). ChIP-qPCR assay showed that site 2 of the TAZ promoter was responsible for binding to NRF2 (Fig. 7F). Then, we performed dual-luciferase reporter assays in BLCA cells, and the results indicated that mutation of the site 2 binding region nearly abolished promoter activity (Fig. 7G). Collectively, these findings confirm that NRF2 regulates TAZ expression, establishing a positive feedback loop between TAZ and NRF2. Furthermore, a series of rescue experiments further supported the notion that this positive feedback loop influences the proliferation of bladder cancer cells by regulating ferroptosis through the GPX4 pathway (Fig. 7H, I). In conclusion, our results indicated that TAZ enhances GPX4-mediated ferroptosis resistance in BLCA cells by establishing a positive feedback loop with NRF2, thereby promoting the malignant progression of BLCA.

## Discussion

Bladder cancer is the most lethal urogenital tumor, characterized by the high rate of incidence, recurrence, and metastasis [27]. Ferroptosis is a regulated form of cell death initiated by reactive oxygen species (ROS), which induce the peroxidation of polyunsaturated fatty acids (PUFAs) in cellular membranes [28, 29]. This process leads to the formation of lipid hydroperoxides, which, in the presence of iron, generate highly reactive lipid radicals [30]. These lipid radicals cause extensive damage to the cell membrane, resulting in the loss of membrane integrity and ultimately triggering cell death [31, 32]. Key regulators of ferroptosis include the lipid peroxidation pathway, iron metabolism, and glutathione peroxidase 4 (GPX4), which is essential for preventing lipid peroxidation and promoting cell survival in bladder cancer [33]. Therefore, elucidating the mechanisms underlying ferroptosis in BLCA is crucial for the development of effective ferroptosis-targeted therapies.

TAZ is overexpressed in various human malignancies and plays a crucial role in tumor initiation and progression [34–36]. Several studies have shown that TAZ regulates ferroptosis and promotes malignant progression in various tumors [37–39]. However, the role of TAZ in inhibiting ferroptosis in BLCA remains unclear. In our study, we analyzed TCGA and GEO BLCA datasets and found that TAZ expression levels in BLCA were significantly higher than in normal tissues. Immunohistochemical staining and Western blot analyses further demonstrated that TAZ was markedly overexpressed in BLCA tissues compared to adjacent normal tissues. Functional experiments demonstrated that TAZ promotes the malignant progression of BLCA cells both in vitro and in vivo. RNA-seq analysis revealed that the expression levels of GPX4 and NRF2 were downregulated in the TAZ-depleted group. GSEA results indicated that TAZ knockdown was closely associated with ferroptosis and glutathione metabolism. Moreover, we treated BLCA cells with various cell death inhibitors, and results suggested that only Fer-1 was able to reverse the inhibitory effect of TAZ knockdown on cell growth. Additionally, ferroptosis-inducing agents Erastin and RSL3 inhibited the growth of TAZ-overexpressing cells. Transmission electron microscopy (TEM) revealed more pronounced mitochondrial damage in TAZ-depleted T24 cells. GPX4 functions as a key target gene directly regulated by NRF2 and plays a crucial role in glutathione metabolism. Recent studies suggest that the NRF2–GPX4 axis is closely associated with ferroptosis [40]. However, further analysis revealed that knockdown of TAZ decreased the mRNA and protein levels of GPX4, while TAZ overexpression resulted in the opposite effect. TAZ depletion decreased GSH levels and increased MDA and ROS levels. Additionally, treatment with RSL3 reduced cell proliferation induced by TAZ overexpression. Consistent with previous studies, our findings indicated that TAZ facilitates BLCA cell proliferation by inhibiting ferroptosis.

To investigate the underlying mechanism by which TAZ regulates ferroptosis, we conducted mass spectrometry analysis and identified NRF2 as a TAZ-interacting protein. Molecular docking models suggested that the TAD domain of TAZ interacts with the Neh1/3 domain of NRF2. Co-immunoprecipitation confirmed the interaction between TAZ and NRF2 under both endogenous and exogenous conditions, and immunofluorescence analysis showed the colocalization of these proteins. Structurally, TAZ consists of the TEAD transcription factor-binding domain (TBD), the transcriptional activation domain (TA), and the WW domain [41, 42], while NRF2 contains seven functional domains (Neh1–7) [24, 43, 44], while Neh3 domain was important for the transactivation activity of NRF2 [45]. As we expected, Co-IP results suggested that the Neh1/3 domain of NRF2 was responsible for the interaction with TAZ, whereas the TA domain of TAZ interacted with NRF2. Dual-luciferase reporter assays indicated that ectopic expression of TAZ significantly enhanced ARE promoter activity, whereas deletion of the TAD domain of TAZ mitigated this effect, which was consistent with previous observations.

Previous studies have extensively reported that the transcription factor NRF2 serves as the main regulatory factor of cellular antioxidant response and can be transcriptionally regulated by TAZ [26, 46, 47]. In this study, TAZ knockdown significantly reduced both NRF2 mRNA and protein levels, subsequently leading to a decrease in the expression levels of NRF2 target genes, including GPX4. Due to its lack of DNA-binding domains, TAZ functions as a transcriptional coactivator that binds to TEAD transcription factors (TEAD1–4) [26, 48]. By analyzing all TEAD family members in the TCGA BLCA dataset, we found that TEAD4 was the most highly expressed in BLCA. Publicly available ChIP-seq data from the Cistrome database (http://cistrome.org/db/#/) revealed that TEAD4 binds to the promoter regions of NRF2. Using the JASPAR database, we identified four potential TEAD4 binding sites on the NRF2 promoter, and ChIP-qPCR and dual-luciferase assays confirmed that TAZ regulates NRF2 at the transcriptional level. Rescue experiments further demonstrated that TAZ inhibits ferroptosis and promotes BLCA cell proliferation through the transcriptional activation of NRF2. Notably, previous studies have shown that NRF2 can also transcriptionally activate TAZ [49]. In our study, NRF2 depletion reduced TAZ mRNA and protein levels, and ChIP-seq data analysis revealed NRF2 binding to the TAZ promoter. ChIP-qPCR and dual-luciferase reporter assays also confirmed that NRF2 regulates TAZ transcriptionally, establishing a positive feedback loop between TAZ and NRF2. This positive feedback loop sustains GPX4 activation and inhibits ferroptosis, thereby promoting the malignant progression of BLCA.

## Conclusion

In conclusion, our results indicated that TAZ was highly expressed in BLCA tissues and promoted malignant progression by inhibiting ferroptosis. Mechanistically, TAZ sustains GPX4 activation and inhibits ferroptosis by establishing a positive feedback loop with NRF2. These insights into the molecular mechanisms governing ferroptosis in BLCA highlight potential novel therapeutic targets for the treatment of bladder cancer.

## Materials and method

### Data acquisition

The transcription profiles of bladder cancer patients were obtained from the TCGA database (https://portal.gdc.cancer.gov/). The BLCA gene microarray expression profiles (GSE13507) were downloaded from the Gene Expression Omnibus (GEO) database (http://www.ncbi.nlm.nih.gov/geo/). Pan-cancer analysis of TAZ was conducted using the online TIMER database (http://timer.cistrome.org/). Additionally, the ChIP-seq data used to predict potential binding sites were acquired from the Cistrome database (http://cistrome.org/db/#/).

### Clinical samples collection

Between 2018 and 2024, samples from BLCA patients were obtained from individuals who had undergone a radical cystectomy at the Second Affiliated Hospital of Nanchang University. Patient tissue samples were collected and processed following approved protocols. Informed consent was obtained from all participants prior to enrollment in the study. This study was approved by the Medical Ethics Committee of the Second Affiliated Hospital of Nanchang University and was conducted in compliance with the principles of the Declaration of Helsinki.

### Total RNA isolation and quantitative RT-PCR

Total RNA was extracted from bladder tissues and BLCA cells using Trizol reagent (TransGen Biotech, Beijing, China), followed by reverse transcription into cDNA with the TransScript First-Strand cDNA Synthesis SuperMix kit (TransGen Biotech, Beijing, China). RT-PCR was performed using qPCR SYBR Green SuperMix (Servicebio, China). Primer sequences used are provided in Table S1.

### Cell lines and cell culture

The bladder cancer cell lines (T24, 5637, BIU-87, UM-UC-3, EJ, J82) and the human uroepithelial cell line SV-HUC-1 were obtained from Procell Life Science and Technology Co., Ltd. (Wuhan, China). The 5637 and BIU-87 cell lines were cultured in RPMI-1640 (Gibco, USA), EJ, UM-UC-3, and J82 in MEM (Gibco, USA), and T24 in DMEM (Gibco, USA). The SV-HUC-1 cells were cultured in Ham’s F-12 K medium (Gibco, USA). All mediums were supplemented with 10% fetal bovine serum (FBS) (Biological Industries, Israel) and 1% penicillin–streptomycin solution (Thermo Fisher, USA). The cells were cultured at 37 °C in a humidified atmosphere containing 5% CO_2_.

### Transfection

The human TAZ shRNA (shTAZ#1: 5’-GCGATGAATCAGCCTCTGAAT-3’, shTAZ#2 5’-GCGATGAATCAGCCTCTGAAT-3’) and NRF2 shRNA (shNRF2#1: 5’- GGUUGAGACUACCAUGGUUTT-3’, shNRF2#2 5’-GCGACGGAAAGAGTATGAGC -3’) were designed and purchased from Hanbio (Shanghai, China). Full-length and deletion constructs of TAZ and NRF2 were also subcloned into pcDNA3.1-3xFlag or pcDNA3.1-HA vectors (Hanbio, Shanghai, China). All constructs were examined by DNA sequencing, and transfections were performed using LipofectamineTM 3000 (ThermoFisher, MA, USA) according to the manufacturer’s protocols. The stable cell lines were screened by treating lentivirus-transfected cells with 5 μg/ml puromycin for 24 h. The transfection efficiency was then confirmed using qRT-PCR and western blot analysis.

### Western blot

Cells and tissues were lysed using RIPA buffer (Beyotime, China), and protein concentrations were determined with a BCA protein assay kit (TransGen Biotech, Beijing, China). Proteins were then boiled in 5× SDS-PAGE loading buffer (New Cell and Molecular Biotech, China) for 5–10 min at 100 °C. Following boiling, proteins were separated by SDS-PAGE and transferred to PVDF membranes (Millipore, Billerica, MA). The membranes were blocked with 5% milk for 2 h, washed three times with TBST, and incubated with primary antibodies overnight at 4 °C. The next day, membranes were incubated with secondary antibodies (anti-mouse or anti-rabbit IgG, Proteintech, China) for 1 h at room temperature. After three additional washes with TBST, the protein bands were detected using a Bio-Rad detection system. The antibodies used in this study are listed in Table S2.

### Cell Counting Kit-8 assay

Cells were plated in 96-well plates at a density of 3000 cells per well and incubated at 37 °C with 5% CO_2_ for 24, 48, 72, and 96 h. At the end of each incubation period, 10 μl of CCK-8 solution (APE, BIO, USA) was added to each well, and the plates were further incubated for 1 h. Absorbance was subsequently measured at 450 nm using a multi-scan spectrophotometer.

### Colony formation assay

For the colony formation assay, 500–1000 transfected cells were seeded in a 6-well plate and incubated for 7–10 days. After the incubation period, the culture medium was discarded and washed three times with PBS, then the cells were fixed with 4% paraformaldehyde for 30 min. Following fixation, the cells were stained with 1% crystal violet for 15 min. Finally, the results were analyzed using ImageJ software.

### Determination of malondialdehyde (MDA) and glutathione (GSH)

For MDA detection, the Malondialdehyde (MDA) Detection Kit (Beyotime, China) was utilized to measure MDA levels in BLCA cells. In cell lysates, MDA reacts with thiobarbituric acid (TBA) to form MDA–TBA adducts, which were quantified by measuring absorbance at 532 nm with a microplate reader and comparing the values to a standard curve. For GSH measurement, the GSH Assay Kit (Beyotime, China) was applied to detect GSH production according to the manufacturer’s protocol. Cell lysates were added to a 96-well plate, followed by detection buffer and GSH-PX working solution. After a 15-min incubation at room temperature, peroxide reagent solution was added, and absorbance was immediately measured at 340 nm to record the initial value. After a further 10-minute incubation, absorbance was measured again. Glutathione peroxidase activity was then calculated following the formula provided in the kit manual.

### Intracellular ROS measurements

The ROS Assay Kit for Superoxide Anion with DHE (Beyotime, China) was used to detect intracellular ROS levels following the manufacturer’s instructions. The cells were incubated with 2 μl of DHE solution at 37 °C in the dark for 20 min, then washed three times with PBS. Finally, the ROS level was detected utilizing DHE by a flow cytometer (FCM, Beckman Coulter, USA) at 535/610 nm, and the results were calculated using FlowJo 10.1 software.

### Co-IP

Lentivirus-transfected cells were lysed in 1 ml of IP buffer (Beyotime, China). The cell lysates were subjected to ultrasonication, followed by centrifugation at 12,000 rpm for 15 min at 4 °C. Twenty-five microliters of the lysate supernatant were reserved as input. The remaining lysate was incubated overnight on a rotating device at 4 °C with Flag magnetic beads (YEASEN, China) and specific antibodies. The magnetic beads were then washed four times, each for 5 min, with cold IP buffer (Beyotime, China). Subsequently, the beads were boiled for 15 min in 25 µl of 1× loading buffer (New Cell and Molecular Biotech, China). After centrifugation, the supernatant was collected, and immunoblotting analysis was performed.

### Chromatin immunoprecipitation (ChIP)

ChIP assay was performed using the SimpleChIP® Plus Enzymatic Chromatin IP Kit (Catalog# 9004, Cell Signaling Technology, USA) following the manufacturer’s instructions. In brief, 293 T cells (4 × 10^6^) were transfected with vector/TAZ or vector/NRF2 plasmids. After transfection, 1% formaldehyde (final concentration) was added to cross-link proteins to DNA. Chromatin immunoprecipitation was carried out using 2 μg of antibodies and Protein G Agarose Beads, followed by overnight incubation at 4 °C with rotation. Finally, quantitative PCR was performed to analyze the results.

### Dual-luciferase reporter assays

The pGL3-ARE-Luc reporters were purchased and designed to assess the transcriptional activity of TAZ and NRF2 via ARE responsiveness (Genechem Bio Company, China). Wild-type and mutant plasmids were generated by Hanbio Bio Company. The Dual-Luciferase Reporter Assay Kit (Hanbio, China) was used to measure the activity of both firefly and Renilla luciferase, following the manufacturer’s instructions.

### Animal model experiments

For the subcutaneous xenograft model, 5 × 10^6^ transfected T24 cells (T24-shNC, T24-shTAZ, T24-Vector, T24-TAZ, T24-shNRF2, T24-shNRF2+TAZ, T24-NRF2, and T24-NRF2+shTAZ) were suspended in 100 μl PBS mixed with Matrigel at a 1:1 ratio and injected into the armpits of nude mice. When the tumor volume reached 800 mm^3^, the mice were sacrificed. Tumor volume was measured daily and calculated according to the following formula: Total tumor volume (mm^3^) = *L* × *W*^2^/2, where “*L*” represents the tumor length and “W” represents the tumor width. All animal experiments were approved by the Animal Care and Use Committee of the Animal Care Committee of the First Affiliated Hospital of Nanchang University (approval No. CDYFY-IACUC-202401QR008).

### Statistical analysis

Continuous variables are presented as mean ± standard deviation (SD), and categorical variables are expressed as numbers and proportions. All statistical analyses and visualizations were conducted using GraphPad Prism (version 9.0). Student’s *t*-test was employed for comparisons between two groups, while one-way and two-way ANOVA were used for analyzing differences among multiple groups. Statistical significance was defined as a *p*-value less than 0.05, with the following annotations: *p* < 0.05 (*), *p* < 0.01 (**), and *p* < 0.001 (***).

## Availability of data and materials

The datasets used and/or analyzed during the current study are available from the corresponding author on reasonable request.

## Supplementary information

Supplementary Figure Legends

Supplementary Figure 1

Supplementary Figure 2

Supplementary Figure 3

Supplementary Figure 4

Supplementary Tables

Original WB data
