# Supplementary material for: TAZ/NRF2 positive feedback loop contributes to proliferation in bladder cancer through antagonistic ferroptosis
Source: Cell Death Discov. 2025 Apr 29;11:208. doi: 10.1038/s41420-025-02506-9 (PMC12041353; doi:10.1038/s41420-025-02506-9)
Supplement: Supplementary file 1 — Supplementary Figure Legends [file 41420_2025_2506_MOESM1_ESM.docx]

**Supplementary Figure Legends**

**Figure S1. TAZ promotes malignant phenotypes of BLCA cells both in vitro and in vivo.** **A-B** qRT-PCR and western blot analyses were performed to confirm the efficiency of TAZ knockdown and overexpression in J82 cells. **C** The CCK-8 assay was conducted to evaluate cell viability in both control and TAZ knockdown cells, as well as in control and TAZ-overexpressing cells. **D** The effect of TAZ expression levels on colony formation in J82 cells was assessed using colony formation assays. *p<0.05, **p<0.01, ***p<0.001.

**Figure S2. TAZ facilitates the proliferation of BLCA cells by inhibiting ferroptosis. A** Western blotting analysis of SLC711A, FLT and TFRC protein levels. **B** Colony formation assays were performed to assess the effects of Fer-1 treatment on colony formation in TAZ-knockdown T24 and 5637 cells. **C** CCK-8 assay was conducted at 24, 48, 72 and 96 h using T24 and 5637 cells from different groups (Vector, TAZ, and TAZ with RSL3). *p<0.05, **p<0.01, ***p<0.001.

**Figure S3. TAZ sustains the activation of GPX4-mediated ferroptosis resistance by establishing a positive feedback loop with NRF2. A-D** qRT-PCR and western blot analyses were performed to confirm the efficiency of NRF2 knockdown and overexpression in T24 and 5637 cells. **E** The subcutaneous xenograft mouse model showed that different groups (Vector, NRF2, and NRF2 with RSL3) affect the growth of BLCA cells in vivo (n=5, P-value<0.05). **F** Tumor volume and **G** tumor weight were determined. **H** Immunofluorescence was implemented to detect the expression levels of NRF2 and Ki67 using tumor tissues harvested from xenograft model mice. *p<0.05, **p<0.01, ***p<0.001.

**Figure S4. TAZ sustains the activation of GPX4-mediated ferroptosis resistance by establishing a positive feedback loop with NRF2. A** Western blot analyses demonstrated that treatment with Verteporfin led to a downregulation of NRF2 and TAZ in protein levels in T24 cells. Colony formation (**B**), CCK8 (**C**), assays indicated that Verteporfin can reverse the effects of NRF2-overexpressing. **D** Western blotting assays suggested that ML385 reversed the effects of TAZ overexpression in T24 cells. Colony formation (**E**), CCK8 (**F**), assays indicated that ML385 reversed the effects of TAZ overexpression in T24 cells. *p<0.05, **p<0.01, ***p<0.001.
