# Supplementary figures and images for: TAZ/NRF2 positive feedback loop contributes to proliferation in bladder cancer through antagonistic ferroptosis
Source: Cell Death Discov. 2025 Apr 29;11:208. doi: 10.1038/s41420-025-02506-9 (PMC12041353; doi:10.1038/s41420-025-02506-9)

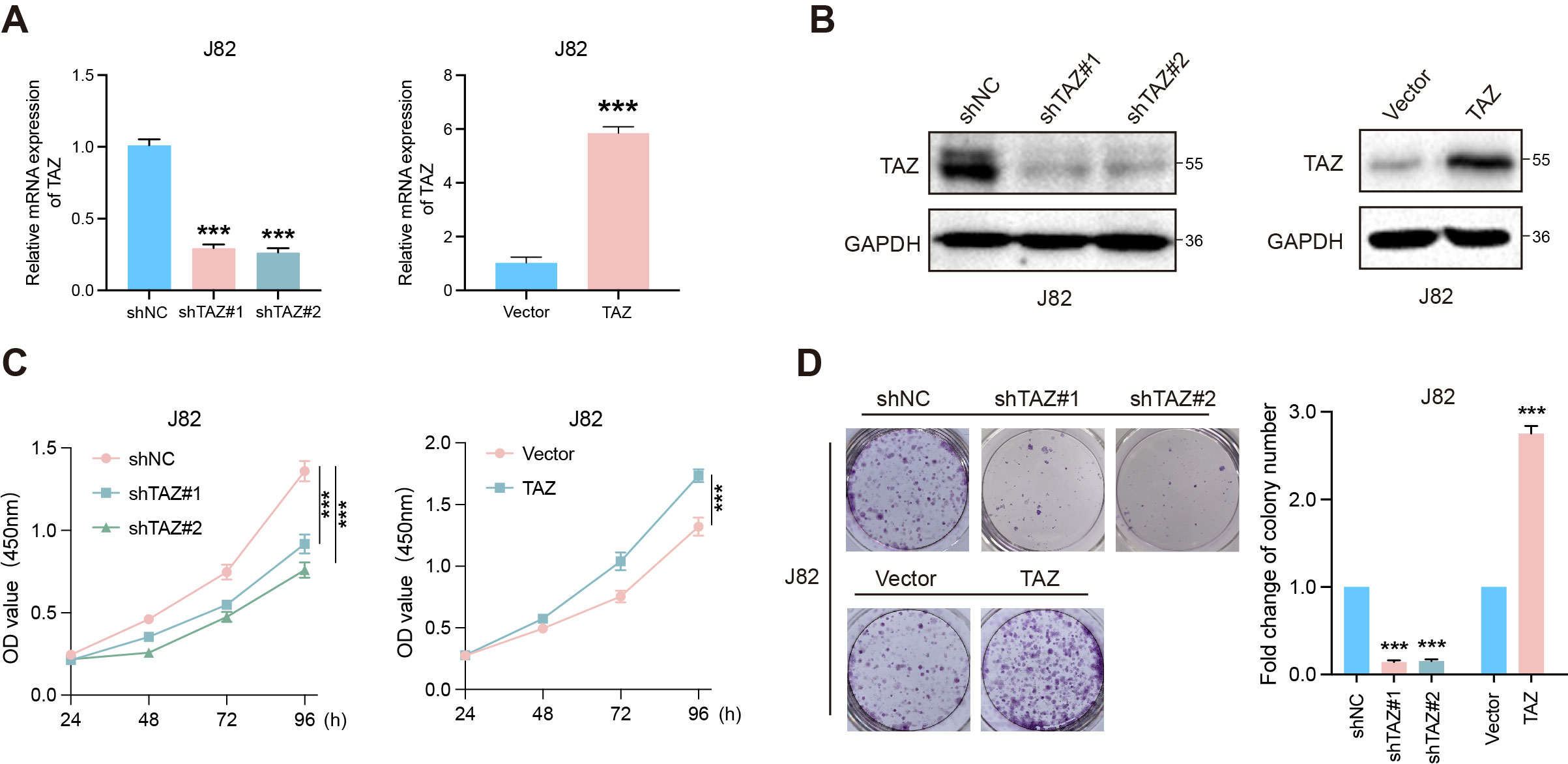

Supplement: Supplementary file 2 — Supplementary Figure 1 [file 41420_2025_2506_MOESM2_ESM.jpg]

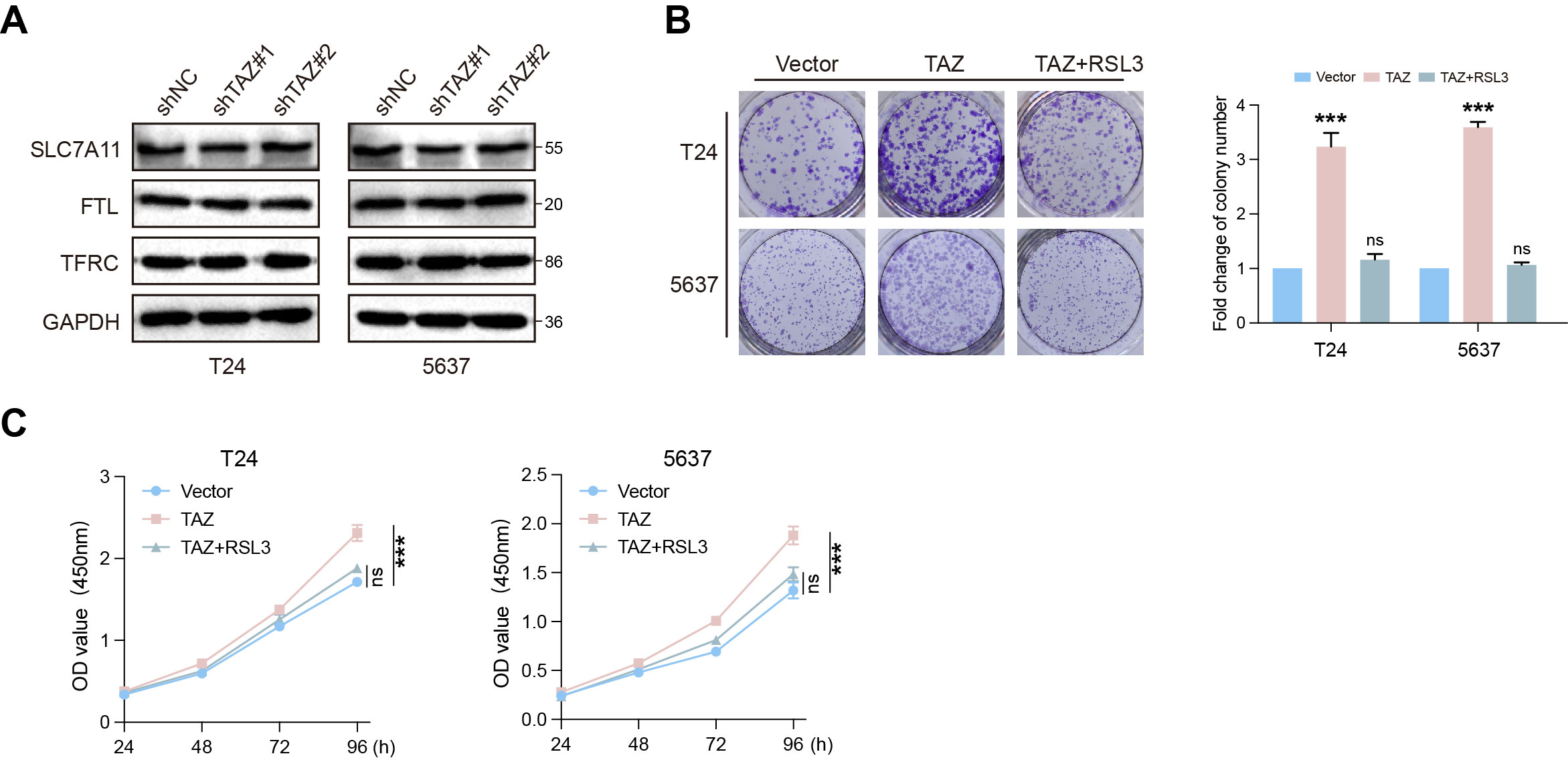

Supplement: Supplementary file 3 — Supplementary Figure 2 [file 41420_2025_2506_MOESM3_ESM.jpg]

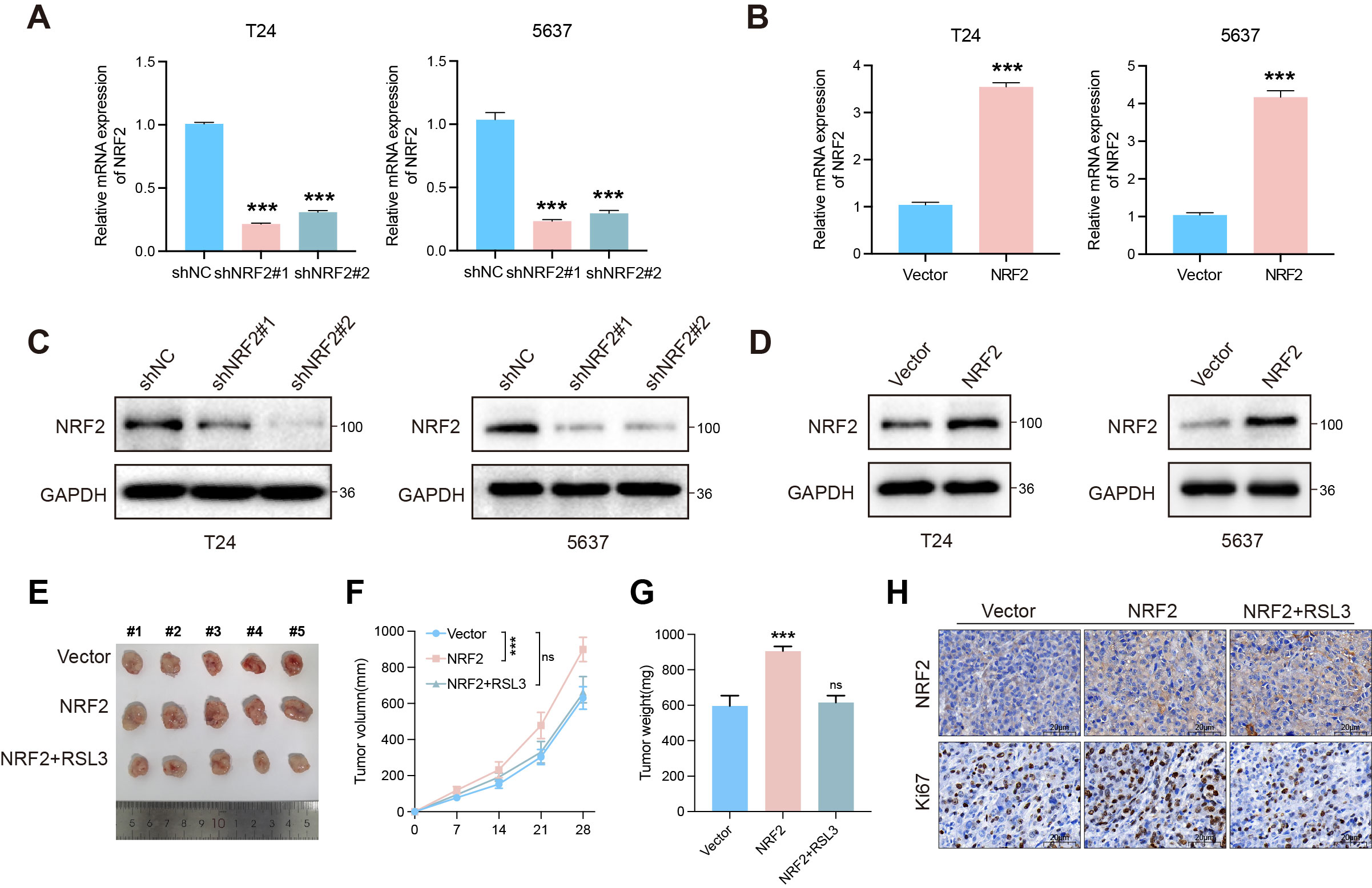

Supplement: Supplementary file 4 — Supplementary Figure 3 [file 41420_2025_2506_MOESM4_ESM.jpg]

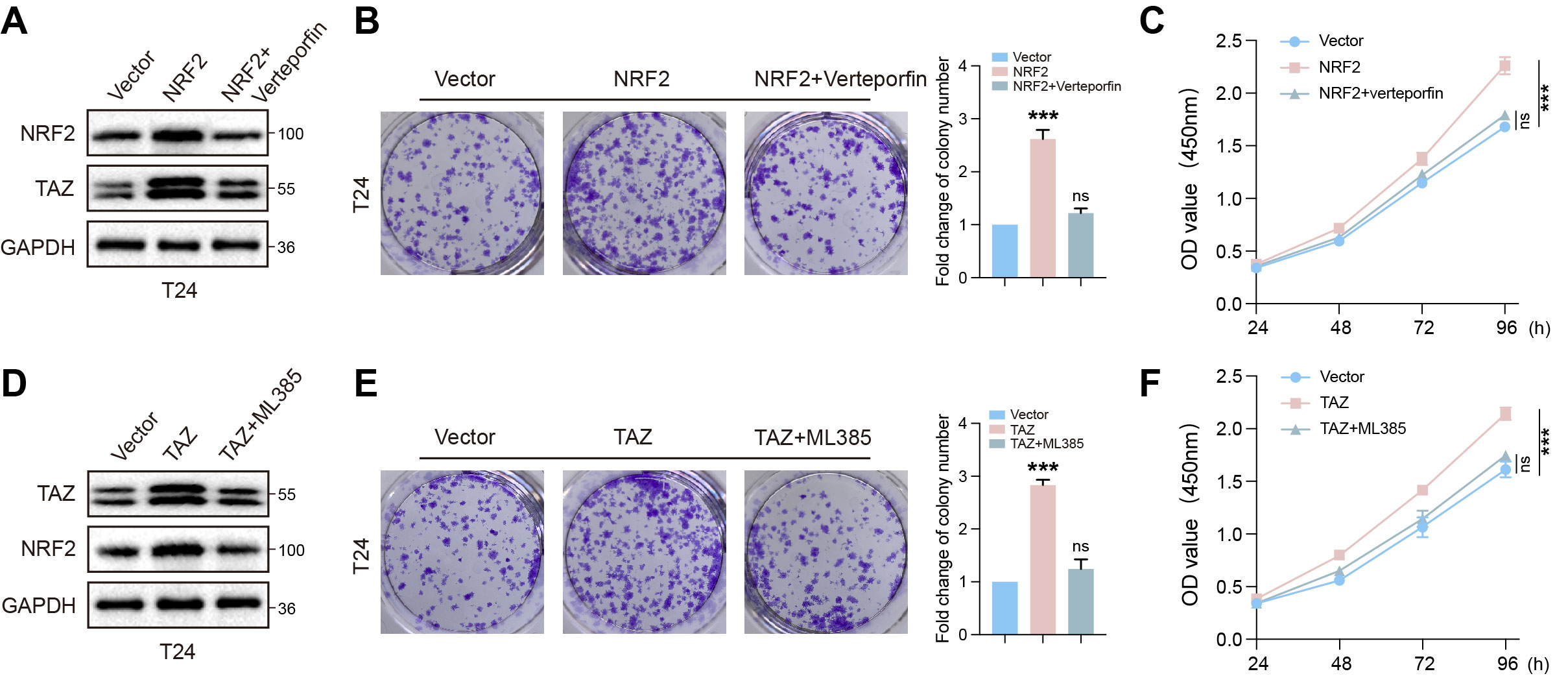

Supplement: Supplementary file 5 — Supplementary Figure 4 [file 41420_2025_2506_MOESM5_ESM.jpg]

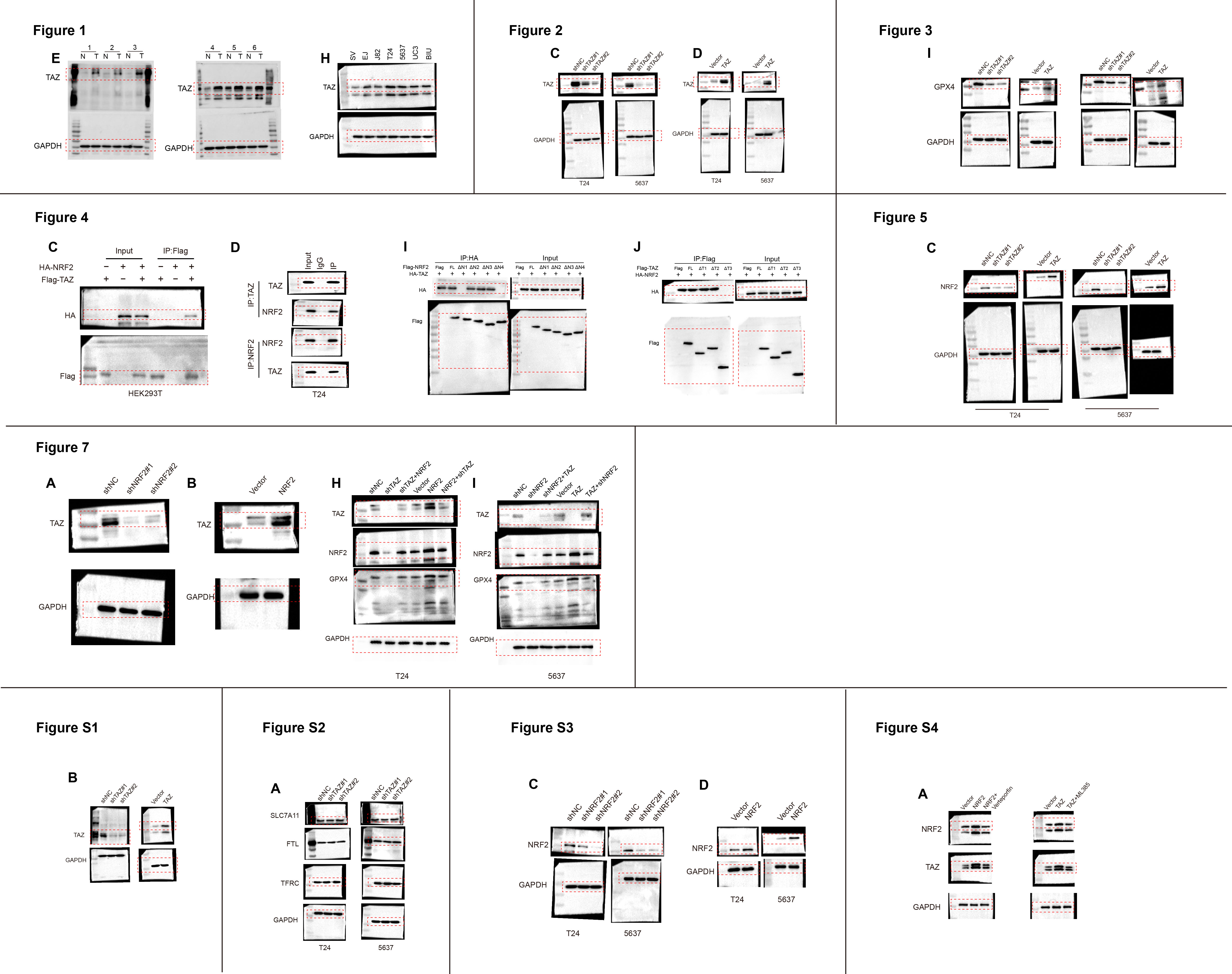

Supplement: Supplementary file 7 — Original WB data [file 41420_2025_2506_MOESM7_ESM.jpg]
