# Supplementary material for: TAZ/NRF2 positive feedback loop contributes to proliferation in bladder cancer through antagonistic ferroptosis
Source: Cell Death Discov. 2025 Apr 29;11:208. doi: 10.1038/s41420-025-02506-9 (PMC12041353; doi:10.1038/s41420-025-02506-9)
Supplement: Supplementary file 6 — Supplementary Tables [file 41420_2025_2506_MOESM6_ESM.docx]

**Supplementary Table**

**Supplementary Table 1.** List of primers of multiple genes was used in this study

| **RT-qPCR** | | |
| --- | --- | --- |
| **Gene (Human)** | **Forward sequence** | **Reverse sequence** |
| TAZ | ACCCGCGAGTACAACCTTCTT | TATCGTCATCCATGGCGAACT |
| NRF2 | CACATCCAGTCAGAAACCAGTG | GGAATGTCTGCGCCAAAAGCT |
| GPX4 | CGATACGCTGAGTGTGGTTTGC | CATTTCCCAGGATGCCCTTG |
| HO-1 | CCAGGCAGAGAATGCTGAGTTC | AAGACTGGGCTCTCCTTGTTGC |
| NQO-1 | ATCACCAGGTCTGCAGCTTC | GCCATGAAGGAGGCTGCTGT |
| GCLC | GATGCCAACGAGTCTGACCA | TGTAAGACGGCATCTCGCTC |
| GAPDH | AGGTGAAGGTCGGAGTCAAC | CGCTCCTGGAAGATGGTGAT |

| **ChIP-qPCR** | | |
| --- | --- | --- |
| **Gene (Human)** | **Forward sequence** | **Reverse sequence** |
| NRF2(Site 1) | AGCTGCTGTTCAAGAGTTATCT | ATAGAGGGCTGTAGACAATTCC |
| NRF2(Site 2) | TGGGAAGTTGCGGGAAGGTCT | ATGGATGACTTCGCAAAGCCG |
| NRF2(Site 3) | TGCTGTCAAGGGTAAGAGTTG | CTCAGGGGAGAAATAATAGCT |
| NRF2(Site 4) | CTTAACATGTTCCCCCTCTTC | CAAAGGATGAGCAGAAACTTC |
| TAZ(Site 1) | TAGGGTTTCCTCTGAGATGGG | CACGTAAACCACTGTGGGGTA |
| TAZ(Site 2) | ACTGTGATGTAGAGCCAGAGC | CGTGATTCCTTGACAGGGTTC |

**Supplementary Table 2.**

List of antibodies and staining conditions for tissue IHC or Western Blot

| Antibody | Catalog # | Vendor | Antigen dilution |
| --- | --- | --- | --- |
| **Primary Antibodies to**: | | | |
| TAZ | 23306-1-AP | Proteintech | 1:1000,1:50***** |
| NRF2 | 16396-1-AP | Proteintech | 1:2000,1:50* |
| GPX4 | 67763-1-Ig | Proteintech | 1:2000,1:50* |
| Ki-67 | ab8191 | Abcam | 1:50* |
| GAPDH | 60004-1-Ig | Proteintech | 1:5000 |
| Flag-Tag | 20543-1-AP | Proteintech | 1:25000 |
| HA-Tag | ab9110 | Abcam | 1:8000 |
| SLC7A11 | 26864-1-AP | Proteintech | 1:1000 |
| TRFC | 10084-2-AP | Proteintech | 1:2000 |
| FTL | 10727-1-AP | Proteintech | 1:2000 |

List of antibodies was used in this study, * For IHC, others for Western Blot
